# Oxygen-Based Adjunct Therapies in Periodontitis: A Systematic Review and Meta-Analysis Within the Framework of Hypoxia and Inflammation

**DOI:** 10.3390/biomedicines14010009

**Published:** 2025-12-19

**Authors:** Tobias Kollmar, Markus Schepers, Andressa V. B. Nogueira, James Deschner, Lena Katharina Müller-Heupt

**Affiliations:** 1 Department of Periodontology and Operative Dentistry, University Medical Center Mainz, Augustusplatz 2, 55131 Mainz, Germany; 2 Institute of Medical Biostatistics, Epidemiology and Informatics, University Medical Center of the Johannes Gutenberg University Mainz, 55131 Mainz, Germany

**Keywords:** hyperbaric oxygen therapy, hypoxia, tissue regeneration, oxygen, ozone, periodontitis

## Abstract

**Background/Aim:** This systematic review and meta-analysis aimed to evaluate the clinical efficacy of oxygen-based adjunct therapies in patients with periodontitis, including ozone therapy, hyperbaric oxygen therapy, and local oxygen delivery, as adjuncts to subgingival instrumentation. These interventions have been proposed to counteract tissue hypoxia and inflammation, which sustain an environment favorable to anaerobic pathogens in periodontitis. **Methods:** An electronic search was conducted in MEDLINE PubMed, the Cochrane Library, the Cochrane Central Register of Controlled Trials, and SciELO. Risk of bias was assessed using the Cochrane Risk of Bias Tool 2. Standardized mean difference was calculated for gains in clinical attachment level, and a random effects model was applied due to high variability. **Results:** The meta-analysis of adjunct ozone therapies presented a pooled standardized mean difference of 0.53 (95% CI [−0.14, 1.19]), indicating a clinically relevant medium effect in favor of ozone therapies, though this effect was not statistically significant and substantial heterogeneity was observed (I^2^ = 70%, *p* < 0.01). Meta-analysis was restricted to adjunct ozone therapies due to the limited availability of qualifying studies for hyperbaric oxygen therapy and local oxygen therapies. **Conclusions:** While the medium effect size in favor of ozone therapies could be clinically relevant, the statistical non-significance underscores the need for more evidence before widespread adoption. Individual studies reported significant benefits for adjunct HBOT and ozonated olive oil, but comparison between oxygen delivery modes was not possible due to heterogeneous protocols.

## 1. Introduction

Periodontitis is a chronic multifactorial inflammatory disease associated with a shift in the oral microbiome—particularly in the subgingival microbiome—towards a dysbiotic biofilm. It is characterized by progressive destruction of the tooth-supporting hard and soft tissues due to chronic inflammation leading to tooth mobility and ultimately to tooth loss if untreated [1,2,3]. In periodontitis, the host immune response is dysregulated and can generate a self-perpetuating pathogenic cycle of dysbiosis and inflammation [4]. Equivalently, the decrease in oxygen concentration with pocket depth and the increase in the prevalence of obligate anaerobic bacterial species occur in such a pathogenic cycle. The oxygen concentration at the base of untreated periodontal pockets measuring 5 mm to 10 mm in depth has been reported to be as low as 1.8%, classifying it as hypoxic [5]. Under low-oxygen conditions, cells may experience metabolic stress and altered mitochondrial function, which can increase the production of reactive oxygen species (ROS). The host’s immune response is characterized by the recruitment of macrophages that release ROS, such as hydrogen peroxide (H_2_O_2_) or superoxide (O^2−^), which are crucial for pathogen elimination [6]. An imbalance between elevated ROS levels and antioxidant defense mechanisms, referred to as oxidative stress, can contribute to further tissue damage and inflammation [7]. Ongoing imbalance can affect the vascular network involved in regulating inflammation, where the hypoxic microenvironment may act as both a cause and a consequence of inflammatory processes [8]. Tissue hypoxia can lead to the activation of genes involved in inflammation, thereby sustaining chronic inflammatory responses [9]. Conversely, chronic inflammation can impair blood flow and oxygen delivery through tissue edema and microvascular abnormalities, worsening hypoxia. Consequently, a synergistic interaction between hypoxia, microbes of the subgingival biofilm, and inflammation may play a pivotal role in the chronicity of periodontitis.

Considering the background and etiology of periodontitis, oxygen therapies have garnered significant interest for their potential to modulate the inflammatory host response including the hypoxic microenvironment within periodontal pockets. Various oxygen-based adjunctive interventions are available, including ozone (O_3_) therapies, hyperbaric oxygen therapy (HBOT) and local oxygen therapies, such as oxygen-releasing biomaterials. Each of these therapies may exhibit distinct variations in terms of oxygen and ROS generation [10] and the subsequent impact on periodontal tissues. Ozone therapy is one potential adjunctive treatment to the gold standard subgingival instrumentation (SI), involving various forms of applying medical-grade ozone gas, generated from medical oxygen, to affected periodontal tissues. It is believed to work through antimicrobial, anti-inflammatory, and immune modulating effects [11]. Ozone can be delivered in its gaseous state, as ozonated water or as ozonated oils such as olive oil or sunflower oil, which act as lipophilic carriers that enable the gradual release of reactive oxygen species [12], potentially prolonging antimicrobial effects in periodontal pockets. Gaseous ozone offers deeper tissue penetration due to its high diffusibility, but requires careful dose control to prevent cytotoxicity. Through HBOT, local hypoxia can be addressed systemically by increasing the amount of dissolved oxygen in the plasma, in addition to achieving optimal hemoglobin saturation. The heightened pressure and increased oxygen concentration are believed to facilitate the healing process and diminish inflammation within the affected tissues [13,14]. It is theorized to assist in tissue repair and to reinforce the body’s innate defenses against infections [14]. Although HBOT has been explored as an adjunct to SI, in the treatment of periodontitis, it is not part of the standard clinical protocols. Existing study protocols typically involve a series of sessions, where once per day, subjects inhale pure oxygen for a total of 90 min while being exposed to a pressure of 2.5 ATA (0.25 MPa) [15].

The aim of this systematic review was to evaluate the clinical effectiveness of currently available oxygen-based adjunctive therapies in the non-surgical treatment of periodontitis. In addition, we sought to summarize the underlying biochemical and pathophysiological mechanisms related to oxygenation and ROS that may contribute to their clinical effects. The relationship between hypoxia and inflammation provides a pathophysiological lens for interpreting the potential role of oxygen-based adjunct therapies in periodontitis. Restoring oxygen tension—whether locally or systemically—may help to rebalance the tissue redox status, modulate inflammatory signaling, and promote tissue repair. Although the included interventions varied in their delivery methods and protocols, we considered it reasonable to analyze them under a common framework due to their shared pathophysiological principle of enhancing tissue oxygenation. To compare the different treatment approaches, we chose clinical attachment level (CAL) as a key indicator of periodontal regeneration for the meta-analysis. While CAL represents a clinically meaningful endpoint reflecting overall periodontal healing, it does not allow differentiation between antimicrobial, anti-inflammatory, or regenerative mechanisms. Therefore, the present findings indicate clinical feasibility rather than mechanistic confirmation of oxygen-mediated modulation of hypoxia or inflammation. In the narrative of our systematic review, we also included studies reporting pocket probing depth (PPD) as a closely related clinical parameter, if CAL was not reported.

## 2. Materials and Methods

Study design and protocol registration: This systematic review and meta-analysis was registered with PROSPERO, the International Prospective Register of Systematic Reviews, under the registration number PROSPERO 2024 CRD42024503973, and adheres to the Preferred Reporting Items for Systematic Reviews and Meta-Analyses (PRISMA) guidelines [16].

Research question: The following PICO framework was used. Population—Subjects with periodontitis; Intervention—Adjunct oxygen therapy to SI; Control—SI only; Outcome—CAL or PPD. CAL and PPD were extracted as primary periodontal outcomes. CAL served as the main endpoint for the quantitative synthesis. PPD values were extracted when CAL was not available and synthesized narratively. A literature search was performed including all studies dealing with this topic. For the comprehensive search strategy, three electronic databases were screened for suitable publications. These sources included the National Library of Medicine, Washington, D. C. (MEDLINE PubMed), the Cochrane Library, the Cochrane Central Register of Controlled Trials, and the Scientific Electronic Library Online (SciELO). All databases were screened for suitable studies until September 4th, 2023. Because the meta-analysis was completed based on this predefined search window, the literature search was not extended beyond this date, and the temporal boundary of the evidence base is acknowledged accordingly.

The literature research strategy was completed using a combination of MeSH terms and free-text keywords. For PubMed, the following terms were used: (((((Ozone) OR (O3)) OR (hyperbaric oxygen)) OR (local oxygen)) OR (hyperbaric oxygen therapy [MeSH Terms])) AND (((((chronic periodontitis [MeSH Terms]) OR (periodontitis)) OR (periodontal disease)))). The search syntax was adapted for each database. No suitable studies have been identified through the search of gray literature.

Inclusion and exclusion criteria were decided by the authors in accordance with PRISMA guidelines. A summary of inclusion and exclusion criteria is provided in Supplementary Table S1. 

Inclusion criteria: In this review, we have chosen to include randomized controlled clinical trials and prospective clinical trials that explored various oxygen therapies in subjects with periodontitis, such as HBOT, ozone therapy, and local oxygen therapy. We set the following detailed inclusion criteria: Prospective studies, including randomized controlled trials and non-randomized controlled trials; Published in English; Inclusion of more than or equal to ten subjects; and Subjects without periodontal therapy in the preceding six months. Studies that did not meet the above-mentioned inclusion criteria were not included.

Exclusion criteria: Literature was excluded if it did not involve the application of oxygen therapy as an adjuvant treatment for periodontal therapy or if study designs included exclusively subjects with systemic diseases (e.g., diabetes mellitus). Reviews, animal studies, in vitro studies, and exclusively microbiological studies were also excluded. In the selection process for the meta-analysis, studies were excluded if the control group did not receive SI or if SI was performed more than once during the study period. Studies were also excluded if the standard deviation (SD) for CAL was not reported or if the reported CAL values were implausible. Furthermore, all studies with adjunct use of antibiotics or unrelated use of antibiotics in the preceding three months were excluded, as well as studies that failed to provide precise exclusion criteria themselves regarding systemic diseases of their respective study group. The time for follow-up had to be at least one month after initial treatment. These criteria were applied to ensure the reliability and comparability of the data across the included studies.

Quality and risk of bias assessment of selected studies: The studies were searched and analyzed by two independent researchers. A quality assessment of all selected full-text articles was performed. For the meta-analysis, randomized, controlled trials were evaluated with the Cochrane Risk of Bias Tool II (RoB 2) using the following criteria: bias arising from the randomization process, bias due to deviations from intended interventions, bias due to missing outcome data, bias in measurement of the outcome, and bias in selection of the reported result. The studies were classified as having a low risk of bias, raising some concerns, or having a high risk of bias in accordance with the Cochrane guidance document [17].

Data extraction and handling of missing data: Outcome data required for quantitative synthesis were only included when standard deviations were reported. No imputations were carried out. Studies with missing, inconsistent, or implausible CAL data were excluded, and all analyses were performed using complete-case data.

Meta-analysis: The Meta-analysis was restricted to adjunct ozone therapies due to the limited availability of qualifying studies for hyperbaric oxygen therapy and local oxygen therapies. Our meta-analysis of adjunct ozone therapies was based on the differences in CAL from baseline to follow-up. To synthesize the data from multiple studies, we conducted a meta-analysis using the R meta package. A random effects model was employed to account for substantial heterogeneity across studies, with the Hartung-Knapp adjustment applied to improve the accuracy of the confidence intervals. We calculated standardized mean differences (Hedges’ g) as the effect size metric using the REML estimator for between-study variance (τ^2^). A forest plot was generated to visualize the pooled effect sizes across studies, sorted by estimated treatment effect.

**Figure 1:**
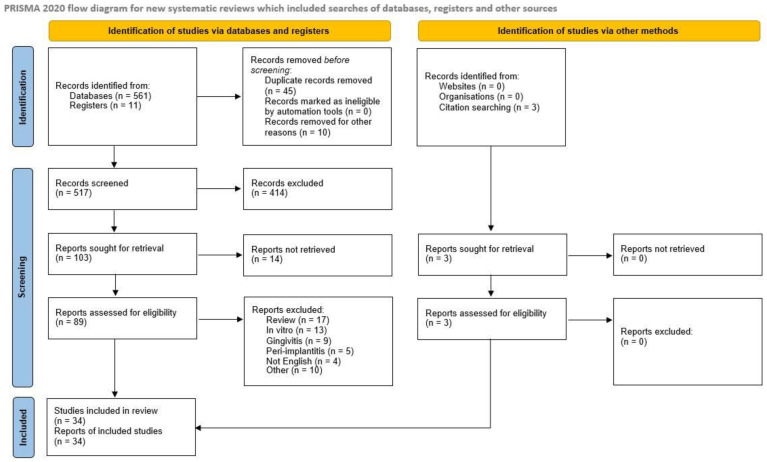
PRISMA 2020 flow diagram.

The complete study selection process is illustrated in the PRISMA 2020 flow diagram (Figure 1). It depicts the number of records identified, included, and excluded, and the reasons for exclusion.

## 3. Results

Evaluation of study quality and risk of bias: Thirty-four randomized controlled or comparative clinical studies were included in the narrative review. Eight randomized controlled trials (RCTs) were found eligible for the meta-analyses; seven for ozone, one for HBOT, and zero for local oxygen therapies. Therefore, a meta-analysis could only be performed for ozone therapies. The study characteristics are summarized in Table 1.

### 3.1. Ozone Therapies

Twenty-six randomized controlled or comparative clinical studies that explored multiple different forms of ozone administration adjunctive to SI were found eligible for inclusion in the narrative of this systematic review. All studies had follow-up periods between one and six months. Six study protocols comprised ozone administration in its gaseous form generated from medical grade oxygen [18,19,20,21,22,23], twelve comprised ozonated water [25,26,27,28,29,30,31,32,33,34,35,36], one was a combination of both [24], six used ozonated olive oil [37,38,39,40,41,42], and one used ozonated sunflower oil [43]. All gaseous ozone studies failed to show significant advantages compared to SI alone, while two of them even reported disadvantageous results, although insignificant [21,22]. Of the ozonated water studies, eight showed better results than SI alone, and five of them significantly [24,25,26,27,28]. Two studies found nonsignificant advantageous results compared to SI + Chlorhexidine (CHX) [32,33] and two studies reported results comparable to SI + photodynamic therapy (PDT) [34,35]. Of the ozonated olive oil studies, only two compared adjunctive administration to SI alone, but showed significant benefits [37,38]. Three studies compared to SI + CHX and found generally comparable results [39,40,41]. Two further studies found nonsignificant advantageous results comparing adjunctive administration of an ozonated olive oil mouthwash and ozonated sunflower oil, respectively, to SI alone [42,43]. Considering that only studies that conducted SI once initially were included, the number of ozone administrations varied substantially. In nine studies, ozone was applied several times, ranging from two to six sessions over the course of two weeks to four months. Regardless of the mode of application, seven of those nine multi-session protocols showed advantageous effects, with five significant ones, while two were not compared to SI alone. The heterogeneity of the study protocols, ozone concentration, and treatment frequencies complicate direct comparison and reduce the overall certainty of the evidence.

### 3.2. Hyperbaric Oxygen Therapies (HBOT)

Six RCTs investigating adjunctive HBOT to SI were found eligible for inclusion into the narrative of this systematic review [44,45,46,47,48,49]. All of them compared SI with adjunctive HBOT to SI alone. Four of them reported statistically significant advantages of adjunctive HBOT over SI alone regarding CAL [44,45,46,47], while one further study confirmed this regarding the closely related PPD [48]. One single study reported a slight, but nonsignificant disadvantage of adjuvant HBOT in CAL [49]. Four study groups used a protocol with ten HBOT sessions, one administered twenty, and one investigated whether eight or sixteen sessions were superior, reporting further improvement in CAL after the second eight sessions, although without statistical significance. Per definition of the Undersea and Hyperbaric Medical Society, a HBOT session comprises 90 min to 120 min of breathing medical grade oxygen (>99.0% oxygen purity) at a pressure of not less than 2.0 ATA (202.65 KPa) [15]. Five of the included studies met this standard with pressures of 2.4 ATA to 2.5 ATA, while one study group administered only 1.4 ATA, and also fell short of the recommended exposure time with only 60 min per session, nevertheless showing the same result of significant benefit of adjuvant HBOT over SI alone [48]. One other study used 72 min of breathing pure oxygen, while four studies used 90 min and 92 min, respectively. Those sessions were suspended for air breaks where subjects breathed the normal atmospheric gas mixture. Neither the number of one to three air breaks, nor their lengths of 5 min to 15 min, affected the overall favorable effect of adjunct HBOT. Follow-up periods were also heterogeneous, with times of final clinical evaluation varying from one to twenty-four months. The two studies that recalled their subjects after one and two years, respectively, found stabilized CAL after one year and deterioration after the second year, although it stayed below baseline values [45,46].

In summary, although a meta-analysis was not feasible, the included HBOT studies collectively indicate beneficial effects of adjunctive therapy. Most studies applied 8–20 sessions of HBOT, with individual sessions lasting 60–120 min at pressures of 1.4–2.5 ATA. Four studies reported statistically significant improvements in CAL, and one study demonstrated significant PPD reduction. Only one study reported a slight, non-significant disadvantage in CAL. Follow-up durations ranged from 1 to 24 months, with long-term data from two studies showing maintained CAL gains after one year and partial relapse by the second year, yet values remained below baseline. These findings suggest that HBOT can enhance short-term periodontal healing, with partial long-term stability, despite heterogeneity in protocols.

### 3.3. Local Oxygen Therapies

One single RCT qualified for inclusion in the narrative of this systematic review, but did not test against SI alone as control group. Medical grade oxygen was locally applied for 15 min at a flow rate of 5 L/min via an individualized elastic silicon tray that covered the whole dental arc sealing tightly to the surrounding mucous membranes. The procedure was repeated three times per day for ten consecutive days. A reduction in CAL was reported, but not significant [50].

### 3.4. Meta-Analysis

#### 3.4.1. Meta-Analysis of Ozone Therapies

**Figure 2:**
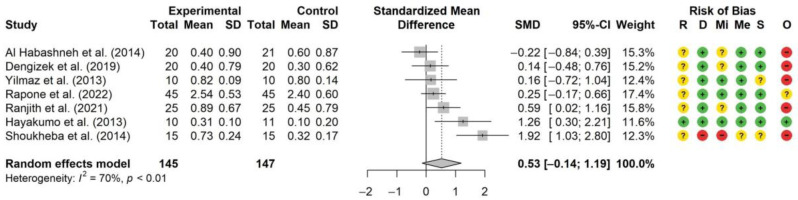
Forest plot of SMD for differences in CAL [mm] from baseline to follow-up across seven ozone therapy studies [18,20,24,25,27,31,37]. RoB 2 graphic is in accordance with Sterne et al. 2019 [17]. R–Bias arising from the randomization process, D–Bias due to deviations from intended interventions, Mi–Bias due to missing outcome data, Me–Bias in measurement of the outcome, S–Bias in selection of the reported result, and O–Overall risk of bias.

Seven studies met the inclusion criteria for the meta-analysis, which was conducted to evaluate the standardized mean difference (SMD) between experimental and control groups across seven studies (Figure 2). The analysis included a total of 145 subjects in the experimental groups and 147 subjects in the control groups. The time points were one to three months after initial treatment.

The pooled SMD was 0.53, with a 95% confidence interval (CI) of [−0.14, 1.19], indicating a medium effect size of the experimental groups compared to the control groups, though this effect was not statistically significant. Individual study effect sizes varied, with SMDs ranging from −0.22 to 1.92. Two studies demonstrated large effect sizes of the experimental groups [25,37]. It should be noted that the largest effect size (SMD = 1.92) was observed in a study with high risk of bias [37], whereas one other study showed a large effect size with a low risk of bias [25]. One study showed a small effect size (SMD = −0.22) in favor of the control group [31]. The heterogeneity among the included studies was substantial, with an I^2^ value of 70% and a statistically significant *p*-value (*p* < 0.01), indicating considerable variability in the effect sizes.

#### 3.4.2. Meta-Analysis of HBOT Therapies

Only one study met the inclusion criteria for a meta-analysis [44]. Therefore, a meta-analysis could not be conducted.

#### 3.4.3. Meta-Analysis of Local Oxygen Therapies

None of the studies met the criteria for inclusion.

## 4. Discussion

One potential approach to interpret our findings considers that different levels of ROS and molecular oxygen may be generated through the various modes of oxygen delivery in periodontal tissues. Excessive ROS concentrations have been associated with both, and with high antimicrobial properties, but also with cytotoxic effects, and therefore they may impair tissue regeneration processes [51], particularly in inflamed or healing periodontal tissues [7,52]. Conversely, adequate reoxygenation of hypoxic periodontal tissues may enhance host defense, reduce anaerobic bacterial load, and promote the resolution of inflammation.

The substantial heterogeneity observed in the meta-analysis of ozone therapies (I^2^ = 70%, *p* < 0.01) indicates considerable variability in effect sizes that may preclude a meaningful single pooled estimate, suggesting that the true effect of ozone therapy likely varies significantly across different contexts and protocols. This aligns partly with the meta-analysis by Deepthi et al. (2020) [53], which reported an overall positive effect despite high heterogeneity (I^2^ ≈ 80%), but without differentiating between different ozone application modes. Gaseous ozone was the mode of administration found to have the least beneficial effect. Only two studies in the meta-analysis applied gaseous ozone [18,20]. Both showed negligible effect sizes, with SMDs of 0.16 and 0.14, respectively. These observations are contrary to the findings of the systematic review by Ambrosio et al. (2023) [54], where gaseous ozone was discussed to be more effective than the aqueous form. In contrast to our review, peri-implantitis was included alongside periodontitis. Mixed study designs and diverse endpoints without a quantitative synthesis could have favored more positive conclusions for gaseous ozone despite substantial protocol heterogeneity. Additionally, it should be noted that gaseous ozone was shown to exert cytotoxic effects on human oral cells in vitro [55], thus potentially impairing periodontal tissue healing. Therefore, only two of six included studies concluded that gaseous ozone therapy may be valuable for periodontal therapy. One study combined gaseous ozone and ozonated water in their protocol and showed a small effect size (SMD = 0.25) [24].

More than half of the studies included in the meta-analysis belong to the ozonated water approach and show a high variation in the effect size (SMDs of −0.22 to 1.26) [24,25,27,31]. This wide variation underscores the substantial heterogeneity in protocols, which complicates direct comparisons and limits the certainty of pooled estimates. Despite this, ozonated water, in comparison to gaseous ozone, has been shown to have a better biocompatibility and less cytotoxicity in vitro [55]. Throughout these studies, there was high variability in the number of treatment sessions, and follow-up periods were relatively short, with the majority ending after one month. One study with a large effect size (SMD = 1.26) used a patented process of making the ozonated water more stable and therefore storable in the form of nano-bubbled ozonated water. Furthermore, this study had a low risk of bias [25]. Eight studies concluded that adjunct use of ozonated water may be valuable for periodontal therapy, one highly recommended its use, and one attributed a highly significant role.

Only one study included in the meta-analysis belongs to the ozonated olive oil approach. It showed a large effect size (SMD of 1.92), but had to be attributed with a high risk of bias, highlighting the potential for inflated estimates. Given the limited and methodologically weak evidence, any conclusions regarding the clinical value of ozonated olive oil should be considered preliminary. While our pooled analysis included this study, future research should aim for rigorous methodology to minimize such biases and ensure the reliability of findings. Furthermore, the subjects were under thirty years of age, and had a diagnosis of high-stage and high-grade periodontitis, and the effect shown at one- and three-month follow-up decreased to a nonsignificant benefit at six months compared to baseline values [37]. From a biomechanical point of view, the ozone is attached to the unsaturated fatty acids of ozonated oils, potentially allowing a more sustained ozone delivery [56,57] and subsequent ROS generation. Furthermore, ozonated olive oil was shown to be highly cytocompatible towards immortalized human gingival fibroblasts in vitro [58]. Unfortunately, none of the study groups compared ozonated olive oil to pure olive oil, so a clear distinction to the additional effects of ozonated olive oil cannot be drawn. Five of seven ozonated olive oil studies concluded that its adjunct administration can be valuable for periodontal therapy. Of all included ozone studies, only one reported adverse effects appearing as dental hypersensitivity [38].

Most consistent significant effects were reported among the HBOT studies. These findings may be related to the highest number of sessions and the feature of systemic effects, such as elevating the amount of oxygen in the bloodstream and consequently in the tissue [59]. Unfortunately, a meta-analysis could not be performed for HBOT, since only two studies tested against SI alone, while only one met all inclusion criteria. Nevertheless, HBOT studies showed a high consistency in treatment protocols among the individual studies. All studies concluded that adjunctive HBOT can harbor therapeutic benefits to periodontal therapy, and one suggested beneficial effects on general health [48]. The relative consistency of HBOT protocols may explain the more reliable outcomes observed compared to other oxygen-based therapies. The resource-intensive nature of HBOT, individual risk factors, and its limited availability reduce its current clinical applicability.

Only one study using local oxygen therapy was included in our review, which was additionally limited by its modalities. Subjects had a diagnosis of necrotizing periodontitis, showing systemic symptoms like fever, and were treated under inpatient conditions. Local oxygen therapy was administered thirty times over the course of ten days combined with systemic antibiotic treatment and a hydrogen peroxide-based mouthwash. Follow-up evaluation was performed on the tenth day, and the adjunct use of local oxygen did show non-significant benefits [50]. Sharing the short retention time inside the periodontal pocket with gaseous ozone and additionally lacking the systemic oxygenation of HBOT, the requirement of numerous administrations becomes clear, which are possible only under inpatient conditions or potentially through home use applications. Given that only a single, highly specific protocol was investigated, the evidence for local oxygen therapy remains extremely limited and inconclusive.

In summary, the limitations of presenting a comprehensive overview of adjunct oxygen therapies were found in short follow-up periods of six months or less; in predominantly small single-center designs; in the unavailability of literature clinically investigating ROS formation in periodontal tissues concerning the different forms of ozone and oxygen application; in the variability of the amount and duration of treatment sessions; in the variability of ozone concentrations generated by the particular devices; in the risk of bias of the individual studies; in our own inclusion and exclusion criteria, that, for example, considered literature published in English exclusively; and in the lack of qualifying studies for an all-encompassing meta-analysis.

Therefore, the strength of the available evidence is limited by the small number of high-quality studies, considerable heterogeneity in protocols and outcome measures, predominantly short follow-up periods, and variable risk of bias. This review applied a predefined search cut-off (4 September 2023), which must be acknowledged as a methodological limitation, as studies published after this date were not captured in the evidence synthesis. These factors collectively reduce the certainty of the current findings and highlight the need for standardized protocols and well-powered, long-term randomized controlled trials to more reliably assess the clinical efficacy and safety of adjunct oxygen therapies in periodontal treatment. Furthermore, future research should complement clinical parameters such as CAL with molecular indicators of hypoxia, inflammation, and regeneration to gain insight into the underlying mechanisms.

## 5. Conclusions

In this systematic review, long-term effects (>12 months) could not be assessed due to lack of available data, but short-term benefits (up to six months) seem feasible, depending on the mode of administration. Significant benefits to SI were particularly observed for HBOT and ozonated olive oil in the narrative synthesis. These findings are based on lower-level evidence and should be interpreted cautiously.

The findings of this meta-analysis showed a potentially clinically relevant medium-sized effect of ozone therapies, although statistically non-significant and with considerable heterogeneity, which therefore should be interpreted with caution. Given the limited number of studies, heterogeneity in treatment protocols, and short follow-up periods, which collectively reduce the certainty of the evidence, future high-quality studies should prioritize longer follow-up periods, ideally exceeding 12 months, to adequately assess the sustained clinical efficacy and long-term stability of periodontal regeneration achieved with adjunct oxygen therapies. The existing evidence, though limited, supports the further investigation of oxygen-based adjunct therapies within the biological context of hypoxia and inflammation that characterizes periodontal disease.

## Figures and Tables

**Table 1:** Characteristics of included studies.

Study Year	Subjects in Test Group Age [Years]	Modality Parameter	Follow-up Administrations Admin. Period	Control Superiority to Control Significance
Yilmaz et al. [18] 2013	10 37–67	gaseous ozone CAL	3 months 5 2 weeks	SI Yes No
Sacco et al. [19] 2017	56 43.3 ± 11.5	gaseous ozone PPD	2 months 1	SI Yes No
Seydanur Dengizek et al. [20] 2019	19 44.7 ± 5.1	gaseous ozone CAL	1 month 1	SI Yes No
Uraz et al. [21] 2019	18 40 ± 6.5	gaseous ozone PPD	3 months 1	SI No n.i.
Skurska et al. [22] 2010	25 39–68	gaseous ozone CAL	2 months 1	SI No No
Tasdemir et al. [23] 2019	36 28–52	gaseous ozone CAL	3 months 1	SI n.i. n.i.
Rapone et al. [24] 2022	45 51.6 ± 14.4	gaseous ozone and ozonated water CAL	6 months 2 2 weeks	SI Yes Yes
Hayakumo et al. [25] 2013	10 45.9 ± 13.8	ozonated water CAL	2 months 1	SI Yes Yes
Pandya et al. [26] 2016	10 20–65	ozonated water PPD	1 month 5 1 week	SI Yes Yes
Ranjith et al. [27] 2022	22 49.2 ± 8.9	ozonated water CAL	1 month 1	SI Yes Yes
Issac et al. [28] 2015	30 35–55	ozonated water CAL	1 month 4 3 weeks	SI Yes Yes
Katti et al. [29] 2013	30 20–60	ozonated water CAL	1 month 6 1 week	SI Yes n.i.
Vasthavi et al. [30] 2020	12 30–65	ozonated water PPD	2 months 1	SI Yes No
Al Habashneh et al. [31] 2015	20 39.7 ± 13.7	ozonated water CAL	3 months 1	SI No No
Kshitish et al. [32] 2010	16 20–60	ozonated water CAL	1 month 1	SI + CHX Yes n.i.
Kaur et al. [33] 2019	20 30–60	ozonated water CAL	3 months 1	SI + CHX Yes n.i.
Ameyaroy et al. [34] 2020	22 45–70	ozonated water CAL	6 months 3 4 months	SI + PDT Yes n.i.
Mehrotra et al. [35] 2023	26 30–65	ozonated water CAL	2 months 2 1 month	SI + PDT No n.i.
Shrivastava et al. [36] 2022	12 30–65	ozonated water PPD	2 months 1	SI Yes No
Shoukheba et al. [37] 2014	15 21–30	ozonated olive oil CAL	6 month 4 3 weeks	SI Yes Yes
Patel et al. [38] 2012	20 30–60	ozonated olive oil CAL	2 month 4 6 weeks	SI Yes Yes
Nambiar et al. [39] 2022	27 38.2	ozonated olive oil CAL	3 months 1	SI + CHX Yes No
Gandhi et al. [40] 2019	25 30–60	ozonated olive oil CAL	3 months 1	SI + CHX No No
Colombo et al. [41] 2021	10 50.0	ozonated olive oil CAL	3 months 1	SI + CHX No No
Nardi et al. [42] 2020	48 30–60	ozonated olive oil PPD	6 months 1	SI Yes No
Tete et al. [43] 2023	15 35–65	ozonated sunflower oil PPD	6 months 1	SI Yes No
Nogueira-Filho et al. [44] 2010	10 37.3	hyperbaric oxygen CAL	3 months 10 5 days	SI Yes Yes
Chen et al. [45] 2002	12 45 ± 5.5	hyperbaric oxygen CAL	12 months 10 n.i.	SI Yes Yes
Chen et al. [46] 2012	30 14–30	hyperbaric oxygen CAL	24 months 10 n.i.	SI Yes Yes
Wandawa et al. [47] 2017	18 30–50	hyperbaric oxygen CAL	1 month 8 (16) 10 (22) days	SI Yes Yes
Burcea et al. [48] 2022	31 48.5 ± 10.5	hyperbaric oxygen PPD	2 months 20 7 weeks	SI Yes Yes
Lombardo et al. [49] 2020	10 18–59	hyperbaric oxygen CAL	3 months 10 10 days	SI No No
Gaggl et al. [50] 2006	15 19–60	local gaseous oxygen CAL	10 days 30 10 days	SI Yes No

Abbreviations: SI = subgingival instrumentation; n.i. = no information; CHX = Chlorhexidine; PDT = photodynamic Therapy; CAL = clinical attachment level; and PPD = pocket probing depth.

## Data Availability

Data is contained within the article or Supplementary Materials.

## Supplementary Materials

The following supporting information can be downloaded at: https://www.mdpi.com/article/10.3390/biomedicines14010009/s1, Table S1: Inclusion and Exclusion Criteria.

## Abbreviations

The following abbreviations are used in this manuscript:

ROS	Reactive oxygen species
HBOT	Hyperbaric oxygen therapy
SI	Subgingival instrumentation
CAL	Clinical attachment level;
PPD	Pocket probing depth
ROB 2	Risk of bias tool II
RCT	Randomized controlled trial
CHX	Chlorhexidine
SMD	Standardized mean difference
SD	Standard deviation
CI	Confidence interval
