# Supplementary material for: Oxygen-Based Adjunct Therapies in Periodontitis: A Systematic Review and Meta-Analysis Within the Framework of Hypoxia and Inflammation
Source: Biomedicines. 2025 Dec 19;14(1):9. doi: 10.3390/biomedicines14010009 (PMC12837386; doi:10.3390/biomedicines14010009)
Supplement: Supplementary file 1 [file biomedicines-14-00009-s001.zip › biomedicines-3987957-supplementary.pdf]

Table S1. Inclusion and Exclusion Criteria.

Category	Criterion	Specification
Narrative Inclusion	Study design	Prospective studies, including randomized controlled trials (RCTs) and non-randomized controlled clinical trials
	Language	Published in English
	Sample size	At least 10 participants per group
	Population	Subjects diagnosed with periodontitis; no periodontal therapy within the preceding 6 months
	Intervention	Adjunctive oxygen-based therapies, including hyperbaric oxygen therapy (HBOT), ozone therapy, or local oxygen therapy, administered in combination with subgingival instrumentation (SI)
	Outcome	Reporting clinical attachment level (CAL) as the primary outcome; probing pocket depth (PPD) if CAL not available
Narrative Exclusion	Population	Study populations exclusive to a certain systemic disease
	Study Design	Microbiological studies
Meta-Analysis Inclusion	Comparator	Control group receiving SI without any adjuvant therapy
	Outcome	Clinical attachment level (CAL) with standard deviation (SD)
	Study design	Prospective studies, including randomized controlled trials (RCTs) and non-randomized controlled clinical trials
	Follow-up	≥ 1 month after initial treatment
Meta-Analysis Exclusion	Intervention	> one session of SI
	Data	Reported CAL values implausible or contradictory
	Antibiotic use	Adjunctive systemic or local antibiotics, or unrelated antibiotic use within the preceding 3 months
	Reporting	Lack of explicit exclusion criteria regarding systemic diseases within the study's own methodology
